# NKT-like (CD3 + CD56+) cells differ from T cells in expression level of cellular protective proteins and sensitivity to stimulation in the process of ageing

**DOI:** 10.1186/s12979-022-00274-z

**Published:** 2022-04-11

**Authors:** Lucyna Kaszubowska, Jerzy Foerster, Zbigniew Kmieć

**Affiliations:** 1 Department of Histology, Medical University of Gdańsk, Dębinki 1, 80-211 Gdańsk, Poland; 2 Department of Social and Clinical Gerontology, Medical University of Gdańsk, Dębinki 1, 80-211 Gdańsk, Poland

**Keywords:** CD3 + CD56+ cells, T lymphocytes, Sirtuin 1, SIRT1, Heat shock protein 70, HSP70, Manganese superoxide dismutase, SOD2, Adaptive stress response, Seniors

## Abstract

**Background:**

NKT-like cells are T lymphocytes coexpressing several NK cell-associated receptors. They are effector lymphocytes of innate and adaptive immunity, and their number increases with age. The study aimed to analyze the expression of cellular protective proteins, i.e. sirtuin 1 (SIRT1), heat shock protein 70 (HSP70) and manganese superoxide dismutase (SOD2) in NKT-like and T cells of the young (‘young’, 31 subjects, age range 19–24 years), seniors aged under 85 (‘old’; 30 subjects, age range 65–84 years) and seniors aged over 85 (‘oldest’, 24 subjects, age range 85–94 years). Both NKT-like and T cells were cultured for 48 h and stimulated with IL-2, LPS and PMA with ionomycin and compared with unstimulated control cells.

**Results:**

The oldest seniors varied from the other age groups by significantly increased expression of SIRT1 and HSP70 in both NKT-like and T cells observed in both stimulated and nonstimulated cells. The analyzed lymphocyte populations of the oldest revealed not only the highest expression of these proteins but also insensitivity to all types of applied stimulation. When NKT-like cells were compared to T cells, higher expression of the studied protective proteins was observed in both stimulated and unstimulated NKT-like cells. Neither CD3 + CD56+ nor CD3+ cells revealed elevated expression of SOD2, and these cells responded to stimulation until very advanced age. T cells revealed higher sensitivity to stimulation with IL-2 regarding SIRT1 and HSP70 expression. NKT-like cells were more sensitive to stimulation with PMA and ionomycin concerning the expression of these proteins. IL-2 did not induce a significant increase in SOD2 expression in the studied age groups.

**Conclusions:**

The oldest seniors developed an adaptive stress response in both T and NKT-like cells regarding the expression of SIRT1 and HSP70, which was increased and insensitive to further stimulation in contrast to SOD2, which showed a more inducible pattern of expression. CD3 + CD56+ cells exhibited higher expression of cellular protective proteins than CD3+ cells in both stimulated and control, nonstimulated cells. NKT-like and T cells showed a distinct sensitivity to the applied stimulatory factors in the respective age groups.

## Background

The process of ageing is associated with progressive disruption of homeostasis resulting from chronic oxidative stress and the development of an imbalance between inflammatory and anti-inflammatory responses [1, 2]. The progressive increase in the proinflammatory status reflected by increased serum levels of the proinflammatory cytokines IL-6 and TNF as well as C reactive protein [3, 4] is caused by a decline in the capacity to cope with numerous stress factors. The ability to activate adaptive homeostasis decreases with age, while its successful maintenance results in extended longevity [5, 6]. The increasing level of oxidative stress that accompanies immunosenescence results from the disparity between the increased generation of reactive oxygen species (ROS) and impaired activity of cellular antioxidative mechanisms [7, 8].

The adaptive stress response provides cell protection against detrimental environmental conditions and various types of injuries [9]. Eukaryotic cells evolved to form numerous networks of signaling pathways to detect and respond to both environmental and endogenous stresses. The key role among these mechanisms is played by chaperones, i.e. a highly conserved family of proteins expressed constitutively or in a stress-inducible manner in both prokaryotic and eukaryotic cells. They are under the control of heat shock factor (HSF-1) and are activated within minutes after the appearance of the stress agent [10]. Chaperones prevent protein misfolding and aggregation, inhibit cell death signaling cascades and preserve intracellular pathways essential for cell survival [11]. HSP70 is one of the chaperones involved in the protein quality control system and regulation of the NF-κB pathway, revealing some modulatory properties [12]. HSP expression was found to correlate with the maximum lifespan of vertebrates, as longer-lived species of mammals or birds revealed higher expression levels of these proteins [13, 14]. Notably, despite the acknowledged role of HSP70 in immunosenescence [15, 16], there are only single studies on HSP70 expression in human lymphocyte subpopulations concerning the ageing process performed on the whole population of lymphocytes [17] or their subsets [18].

Heat shock factor is one of the transcription factors modulated by sirtuin 1, which is the most conserved mammalian (NAD+)-dependent histone deacetylase known to deacetylate a number of proteins involved in various cellular pathways concerning the stress response, apoptosis and cell growth [19]. Sirtuin 1 also modulates the functions of numerous receptors and regulatory proteins PPAR-γ, PGC-1α, AMPK, eNOS and transcription factors, including p53, NF-κB, FOXO and E2F1 [20]. Forkhead box O (FOXO) transcription factors (FOXO1, FOXO3a and FOXO4) are targets of SIRT1 and are especially important for cell survival, as they are involved in the activation of ROS-detoxifying enzymes, including SOD2 and catalase [21, 22]. The protective function of SIRT1 results from deacetylation of p53, which prevents its transcriptional activity and inhibits p53-dependent apoptosis [23].

Moreover, SIRT1 activates the FOXO1a transcription factor, which in turn prevents oxidative stress by upregulating catalase activity [24]. Studies performed on model organisms, i.e. yeast [25], worms [26], flies [27] and mice [28] showed that sirtuins may be regarded as evolutionarily conserved mediators of longevity. Finally, Kilic and coworkers analyzed SIRT1 levels in the sera of people of different ages and found a major increase in sirtuin protein expression in older people and a significant positive correlation between SIRT1 levels and age in the studied population [29].

Reactive oxygen species (ROS) are byproducts of aerobic respiration and can damage numerous cellular macromolecules, including lipids, proteins and nucleic acids. This damage, according to the free radical theory of ageing, contributes enormously to the process of cell senescence [30, 31]. Superoxide dismutases, including mitochondrial manganese superoxide dismutase, are involved in the decomposition of ROS and the limitation of oxidative stress, resulting in the longer lifespan observed in yeast, *Caenorhabditis elegans*, fruit flies and mammalian cells [32]. The overexpression of SOD2 in budding yeast *Saccharomyces cerevisiae* extended the lifespan by 30% [33] and in *C. elegans* by 50% [26]. When SOD2 was overexpressed in *Drosophila melanogaster*, the mean lifespan of the flies increased by an average of 16% [34]. A similar phenomenon was described in a mouse model in which a small, 4% increase in the mean lifespan of SOD2 transgenic mice was observed; however, it reached 18% in long-lived animals [35].

Despite the well-established role of SIRT1, HSP70 and SOD2 in the process of ageing [22, 36–38], little is known about their involvement in the ageing of NKT-like cells, which number increases with age, similar to NK cells [39]. NKT-like cells constitute approximately 5–15% of the peripheral T cell population [40]. Innate-like T cells or NKT-like cells phenotypically are characterized by the coexpression of TCRs with NK-associated receptors (NKRs), such as CD56, CD161, CD16, CD94 and CD57 [41], in contrast to classical NKT (“invariant NKT”; iNKT) cells expressing invariant TCRs (Va24Ja18) that recognize glycolipids presented by the CD1d molecule. Invariant NKT cells account for only 0.1–1% of T cells in human peripheral blood [42]. Both subsets are also differentially affected by ageing since the frequency of peripheral blood classical NKT cells decreases in the elderly, contrary to nonclassical NKT cells, i.e. NKT-like cells [43].

Research on cellular protective proteins, including SIRT1, HSP70 and SOD2, performed so far on human peripheral blood lymphocytes showed the increased expression of these proteins in the oldest seniors both in NK cells [44] and in T and NKT-like cells [45] from whole blood samples analyzed shortly after their collection. The elevated expression of these cellular protective proteins was also maintained in NK cells cultured for 48 h [46]. Then, the effects of stimulation with IL-2, lipopolysaccharide (LPS) and PMA with ionomycin on the expression levels of SIRT1, HSP70 and SOD2 in NK cells [46] and their subpopulations, i.e. CD56dim and CD56bright cells in different age groups have been described [47].

The stimulating agents applied in the study are involved in different signaling pathways initiating the process of lymphocyte activation. Interleukin 2 is a major growth factor for T lymphocytes and NK cells [48]. This cytokine also stimulates the synthesis of pro- and anti-inflammatory cytokines in NKT cells [49] and is associated with the process of their activation [50]. LPS is a component of the outer membrane of gram-negative bacteria and is known to activate cells of both innate [51] and adaptive immunity [52] via the Toll-like receptor (TLR) signaling pathway. LPS is a ligand for TLR4 receptors, which are expressed on the surface of antigen presenting cells (APCs) but also on the cell membrane of NK cells, NKT cells and T lymphocytes [53]. Phorbol 12-myristate 13-acetate (PMA) is a protein kinase C (PKC) activator used for the strong and nonspecific stimulation of both T lymphocytes [54, 55] and NK cells [55, 56] in combination with ionomycin, a calcium ion channel-opening antibiotic that mimics the action of IP3, thereby increasing the Ca2+ concentration in the cytoplasm [57].

Although the expression levels of SIRT1, HSP70 and SOD2 in the elderly and the oldest seniors were described earlier by our group in T lymphocytes and NKT-like cells from whole blood samples and compared with the levels of these proteins in the young [45], the impact of stimulatory agents on the expression of these protective proteins in CD3 + CD56+ and CD3+ cells of the young, old and oldest seniors was not reported. Therefore, the aim of the study was to analyze the expression of SIRT1, HSP70 and SOD2 in both nonstimulated and stimulated with IL-2, LPS and PMA with ionomycin T (CD3+) lymphocytes and NKT-like (CD3 + CD56+) cells of the young, elderly and oldest seniors. The control, unstimulated CD3+ and CD3 + CD56+ cells were also cultivated 48 h in vitro, similar to stimulated with various factors T and NKT-like cells.

## Methods

### Participants

Eighty-five volunteers aged between 19 and 94 years (63 women and 24 men) participated in the study. The exclusion criteria included CRP > 5 mg/L, cancer, autoimmune disease, diabetes, infection, use of immunosuppressors, glucocorticoids or nonsteroidal anti-inflammatory drugs (NSAIDs) and moderate to severe dementia assessed using the “Mini Mental State Examination” (MMSE below 23 points) [58]. The geriatric conditions of senior volunteers were also considered by applying Katz’s scale to assess “activities of daily living” (ADL), and only seniors with 5–6 points were enrolled in the study [59]. Senior volunteers were recruited among inhabitants of local senior houses, and young volunteers were students of Medical University of Gdańsk, Poland. The participants were subdivided into 3 groups: 31 young subjects in the study referred to as ‘young’ (mean age 20.9 ± 0.2 years, range 19–24 years, 22 women and 9 men); 30 seniors aged under 85 referred to as ‘old’ (mean age 75.6 ± 0.6 years, range 65–84 years, 20 women and 10 men) and 24 seniors aged over 85 referred to as ‘oldest’ (mean age 88.5 ± 0.3 years, range 85–94 years; 19 women and 5 men). All volunteers signed informed consent forms, and the study received approval from the Ethical Committee of the Medical University of Gdańsk, Poland (225/2010). The immunological characteristics of the study population were described earlier [44, 45]. Briefly, all studied age groups presented normal white blood cell (WBC) counts and CRP (C reactive protein) values and the groups did not differ regarding the number and percentage of lymphocytes, although some significant differences concerning CRP values and WBC counts were observed between seniors and the young individuals [44]. All compared groups did not differ in the number of NKT-like cells, however, seniors under 85 revealed the highest percentage of these cells, whereas the young had significantly more T cells in one microliter of peripheral blood and presented a significantly higher percentage of CD3+ cells in the population of lymphocytes [45].

### Preparation of peripheral blood mononuclear cell cultures

Peripheral blood mononuclear cells (PBMCs) were isolated from venous blood samples collected in tubes with EDTA by conventional Ficoll-Uropoline density gradient centrifugation. PBMCs were then washed and resuspended in RPMI 1640 medium supplemented with 5% FBS, penicillin (100 U/ml) – streptomycin (100 μg/ml) and 2-mercaptoethanol (5 × 10^− 5^ M) (all purchased from Sigma - Aldrich, Saint Louis, MO, USA). Cells (5 × 10^5^ / 0.5 ml) were cultured for 48 h in the absence (control) or presence of IL-2 (100 U/ml) (BD Biosciences, San Jose, CA, USA), LPS (1 μg/ml) or PMA (50 ng/ml) and ionomycin (500 ng/ml, all purchased from Sigma-Aldrich). PBMCs treated in this way were studied for the expression of SIRT1, HSP70 and SOD2.

### Staining of surface and intracellular antigens for flow cytometry

PBMCs (2.5 × 10^5^ cells) were aliquoted into flow cytometry tubes, and CD3-PE-Cy7-conjugated (0.125 μg/ml; clone SK7) (BD Biosciences, San Jose, CA, USA) and then CD56-APC-conjugated (0.6 μg/ml; clone NCAM16.2) (BD Biosciences, San Jose, CA, USA) monoclonal antibodies were added for cell surface antigen staining. After 30 min of incubation in the dark at room temperature, the cells were washed twice with 1 ml of BD Staining Buffer (PBS without Ca^2+^ and Mg^2+^, 1% FBS, 0.09% sodium azide) and resuspended in 0.25 ml of Fixation/Permeabilization Solution for 20 min at 4 °C following the manufacturer’s protocol (BD Cytofix/Cytoperm Fixation/Permeabilization Kit). Then, the cells were washed twice with 1 ml of BD Perm/Wash buffer, and relevant volumes of MnSOD-FITC-conjugated (1 μg/ml; clone MnS-1) (eBioscience, San Diego, CA, USA), Hsp70-PE-conjugated (1 μg/ml; clone N27F34) (Abcam, Cambridge, England) and SIRT1-Alexa Fluor 488-conjugated (1 μg/ml; clone 19A7AB4) (Abcam, Cambridge, England) monoclonal antibodies were added for staining of intracellular antigens following the manufacturer’s instructions. After 30 min of incubation in the dark at room temperature, the cells were washed twice with 1 ml of BD Perm/Wash buffer and resuspended in Staining Buffer prior to flow cytometric analysis. Samples were run on a BD FACSCalibur flow cytometer equipped with an argon-ion laser (488 nm), and data were evaluated with BD CellQuest Pro software (BD Biosciences, San Jose, CA, USA) after collecting 10,000 gated events (lymphocytes). Peripheral blood lymphocytes were gated using forward (FSC) and side scatter (SSC) parameters. Then, CD3+ and CD3 + CD56+ subsets were analyzed for the frequency of cells expressing particular cellular protective proteins (SIRT1, HSP70, SOD2). Relevant isotype controls were also applied. The results were then presented in two ways as percentages of CD3+ and CD3 + 56+ cells with the expression of the studied protein (% of positive cells) to detect changes in the composition of the analyzed cell populations and the relative expression, i.e. mean fluorescence intensity (MFI) to assess changes in target protein expression.

### Statistics

All data are expressed as the mean ± SEM. The normality of the data distribution was analyzed by the Shapiro-Wilk test. The Kruskal-Wallis test was used to compare experimental data for nonparametric distribution in the analysis of three independent age groups. The Mann-Whitney U test for nonparametric distribution was applied to compare two independent samples, and the Wilcoxon signed-rank test for nonparametric distribution was used to compare two related samples. The Spearman correlation coefficient (Rs) was applied to present the strength of the relationship between variables (Statistica, version 13; Statsoft, Tulsa, OK, USA). Differences or correlations with *p* < 0.05 were considered statistically significant.

## Results

### Expression of SIRT1 in nonstimulated and stimulated CD3+ and CD3 + CD56+ cells of the studied age groups

**Fig. 1:**
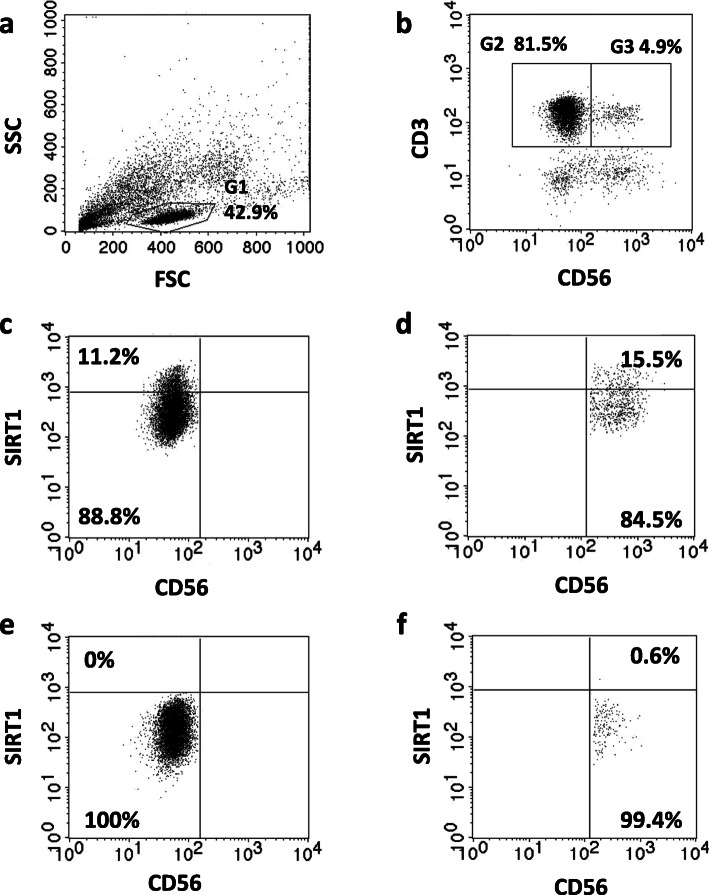
Gating strategy performed for flow cytometric analysis of T lymphocytes (CD3+ cells) and NKT-like cells (CD3 + CD56+ cells). **a.** Lymphocyte gating in the population of PBMCs – lymphocytes were defined as FSC_low_/SSC_low_ cells (G1). **b.** T and NKT-like cell gating – T lymphocytes were defined as the CD3+ positive population (G2), and NKT-like cells were defined as CD3 + CD56+ positive cells (G3). **c.** T cells expressing SIRT1 were identified in the upper left quadrant (Q1). **d.** NKT-like cells expressing SIRT1 were identified in the upper right quadrant (Q2). **e.** Isotype control for SIRT1-positive T cells. **f.** Isotype control for SIRT1-positive NKT-like cells. The analysis of HSP70 and SOD2 expression was performed in the same way. All age groups were analyzed with the same gating strategy

The gating strategy performed for flow cytometric analysis is demonstrated in Fig. 1. The results of flow cytometry experiments are presented as the percentages of CD3+ or CD3 + CD56+ cells revealing the expression of the studied protein (% of positive cells) and the relative expression, i.e. mean fluorescence intensity (MFI) measured in the samples.

**Fig. 2:**
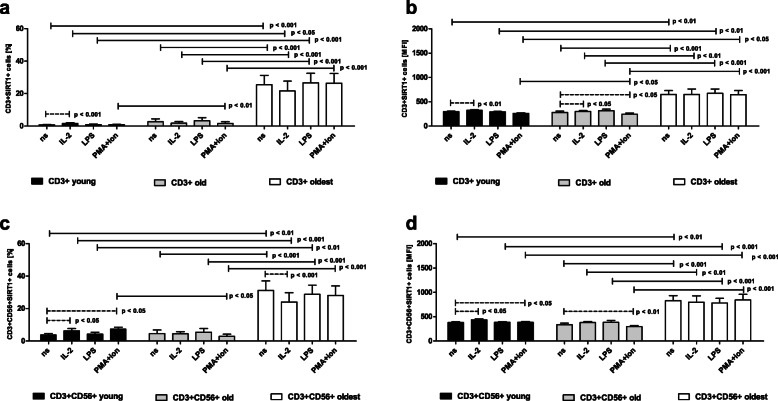
Expression of SIRT1 in nonstimulated and stimulated with IL-2, LPS and PMA/ionomycin CD3+ and CD3 + CD56+ cells of the young, old and oldest age groups. Data are presented as the mean ± SEM and show the expression of sirtuin 1 in the studied cell populations demonstrated as the percentage of cells with the expression of SIRT1 protein (%) and mean fluorescence intensity (MFI). Solid horizontal lines denote statistically significant differences found between similarly treated cells of different age groups, i.e. young vs. old, young vs. oldest and old vs. oldest. Dashed horizontal lines above paired bars denote statistically significant differences between nonstimulated vs. stimulated cells within the same age group. **a.** Expression of SIRT1 in CD3+ cells (%). **b.** Expression of SIRT1 in CD3+ cells (MFI). **c.** Expression of SIRT1 in CD3 + CD56+ cells (%). **d.** Expression of SIRT1 in CD3 + CD56+ cells (MFI)

The expression of SIRT1 in unstimulated CD3+ cells of the young was rather low, i.e. 0.74 ± 0.28% of the analyzed cells, with no significant differences observed between these cells and cells stimulated with LPS and PMA with ionomycin. CD3+ cells of the young stimulated with IL-2, however, presented a twofold increase in SIRT1 expression (Fig. 2a). The same increase was found in the corresponding MFI analysis (Fig. 2b). Interestingly, the expression of SIRT1 in nonstimulated CD3 + CD56+ cells of the young was significantly higher than in T cells and concerned 3.75 ± 0.96% of the analyzed cells (*p* < 0.001). These cells also revealed higher sensitivity to stimulation. They responded to stimulation with IL-2 and PMA with ionomycin, revealing a nearly twofold increase in the expression of this protective protein in both samples (Fig. 2c). These increases were also observed when the relative expression of SIRT1 was analyzed (Fig. 2d).

The percentage of unstimulated CD3+ cells expressing SIRT1 in the old group was slightly higher than that in the young group (2.62 ± 1.8%), however this difference was not statistically significant. No significant differences were observed between these control cells and cells stimulated with IL-2, LPS or PMA with ionomycin (Fig. 2a). In contrast, the analysis of the relative expression of SIRT1 in CD3+ cells of the old showed some sensitivity to stimulation with IL-2 (mild increase). However, stimulation with PMA and ionomycin resulted in a mild decrease in SIRT1 expression (Fig. 2b). The expression of SIRT1 in nonstimulated CD3 + CD56+ cells of the old group was significantly higher than that in T cells and occurred in 4.59 ± 2.26% of cells (*p* < 0.05). These cells also showed no sensitivity to the process of stimulation (Fig. 2c). However, similar to T lymphocytes, MFI analysis showed a mild decrease in SIRT1 expression in CD3 + CD56+ cells stimulated with PMA/ionomycin (Fig. 2d). A related tendency, which was not significant, was found in CD3 + CD56+ cells treated in the same way in the analysis of percentages (Fig. 2c).

The expression of SIRT1 in unstimulated CD3+ cells of the oldest seniors was enormously elevated, i.e. 25.43 ± 5.7% of the analyzed cells. Interestingly, this rather high and constant expression also characterized the cells stimulated with IL-2, LPS and PMA with ionomycin, as no significant differences were observed between the studied cells (Fig. 2a). These results were also confirmed in the analysis of the relative expression (Fig. 2b). Interestingly, nonstimulated CD3 + CD56+ cells of the oldest age group showed higher SIRT1 expression than T cells, which involved 31.12 ± 5.98% of cells, although this increase was not statistically significant. Similar to T lymphocytes, this expression was largely constant and insensitive to the process of stimulation (Fig. 2c) and was also confirmed by MFI analysis (Fig. 2d) with the exception of NKT-like cells stimulated with IL-2 presenting a decrease in SIRT1 expression in percentage analysis (Fig. 2c), although it was not confirmed in MFI analysis (Fig. 2d).

To summarize, the oldest seniors presented the highest expression of SIRT1, and this pattern was similar for both T and NKT-like cells, elevated and rather constant. T cells revealed higher sensitivity to activation with IL-2 than NKT-like cells, as they responded to IL-2 stimulation by increased SIRT1 expression not only in the young (% and MFI analysis) but also in the old (MFI analysis), in contrast to NKT-like cells, which increased SIRT1 expression only in the young (% and MFI analysis). CD3 + CD56+ cells were more sensitive to stimulation with PMA and ionomycin that induced the increase in SIRT expression in the young group (% and MFI analysis) in contrast to CD3+ cells, which did not respond to PMA stimulation by increasing expression of this sirtuin. The expression of SIRT1 was significantly higher in NKT-like cells than in T cells in the young and old age groups and revealed a similar tendency, although not statistically significant, in the oldest senior group in both types of analysis.

### Expression of HSP70 in nonstimulated and stimulated CD3+ and CD3 + CD56+ cells of the studied age groups

**Fig. 3:**
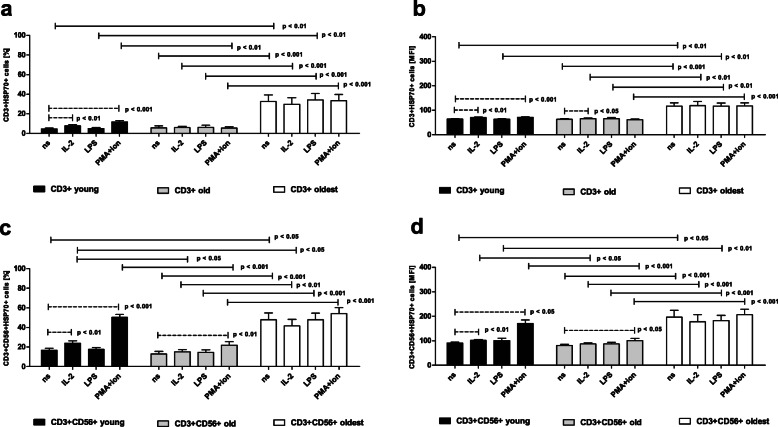
Expression of HSP70 in nonstimulated and stimulated with IL-2, LPS and PMA/ionomycin CD3+ and CD3 + CD56+ cells of the young, old and oldest age groups. Data are presented as the mean ± SEM and show the expression of HSP70 in the studied cell populations demonstrated as the percentage of cells with the expression of HSP70 protein (%) and mean fluorescence intensity (MFI). Solid horizontal lines denote statistically significant differences found between similarly treated cells of different age groups, i.e. young vs. old, young vs. oldest and old vs. oldest. Dashed horizontal lines above paired bars denote statistically significant differences between nonstimulated vs. stimulated cells within the same age group. **a.** Expression of HSP70 in CD3+ cells (%). **b.** Expression of HSP70 in CD3+ cells (MFI). **c.** Expression of HSP70 in CD3 + CD56+ cells (%).**d.** Expression of HSP70 in CD3 + CD56+ cells (MFI)

Similar to SIRT1, the expression of HSP70 in unstimulated CD3+ cells of the young group was rather low and involved 4.68 ± 0.83% of the analyzed cells. These cells, however, responded to stimulation with IL-2 and PMA with ionomycin and showed a 1.7-fold increase and over 2.5-fold increase in the expression of HSP70, respectively (Fig. 3a). These increases were also observed in the corresponding MFI analysis (Fig. 3b). The nonstimulated CD3 + CD56+ cells of the young group revealed significantly higher expression of HSP70, which involved 16.71 ± 1.9% of the analyzed cells (*p* < 0.001). Similar to T lymphocytes, these cells responded to stimulation with IL-2 and PMA/ionomycin and this response showed a 1.4-fold and over threefold increase in HSP70 expression, respectively (Fig. 3c). These increases were also confirmed in the analysis of the relative expression (Fig. 3d).

In the old age group, the expression of HSP70 in unstimulated CD3+ cells was slightly higher than that in the young, however this difference was not statistically significant. It occurred in 5.62 ± 2.09% of cells, which were insensitive to any type of stimulation (Fig. 3a). These results were confirmed by MFI analysis with the exception of cells stimulated with IL-2, which revealed a mild increase in the expression of this protective protein (Fig. 3b). The same tendency, however, without statistical significance was also observed in CD3+ cells stimulated with IL-2 in the percentage analysis (Fig. 3a). CD3 + CD56+ cells of the old group presented significantly higher than in CD3+ cells expression of HSP70 in control, unstimulated cells; i.e. 12.96 ± 2.59% of the analyzed cells (*p* < 0.001). In contrast to T cells, NKT-like cells presented sensitivity to stimulation with PMA/ionomycin, which corresponded to a nearly twofold increase in the percentage of cells revealing HSP70 expression (Fig. 3c), and this increase was also observed in the MFI analysis (Fig. 3d).

The oldest seniors were characterized by the highest expression of HSP70 in nonstimulated T lymphocytes found in 32.69 ± 6.56% of cells. Similar to SIRT1 expression, these cells were insensitive to any type of stimulation (Fig. 3a), and the results were confirmed by MFI analysis (Fig. 3b). CD3 + CD56+ cells of the oldest seniors showed higher expression of HSP70 than CD3+ cells, which involved 47.86 ± 6.89% of cells, although this increase was not statistically significant. Similar to T cells, this expression was rather constant and insensitive to any type of stimulation (Fig. 3c). Analogous observations were made in the corresponding analysis of the relative expression (Fig. 3d).

In summary, the analysis of HSP70 expression showed that the oldest seniors presented the highest expression of this protective protein, and this pattern was similar for both T and NKT-like cells, elevated and rather constant, analogous to SIRT1 protein. T cells revealed higher sensitivity to stimulation with IL-2 than NKT-like cells, as they responded to IL-2 stimulation both in the young (% and MFI analysis) and the old (MFI analysis) and NKT-like cells only in the young (% and MFI analysis). CD3 + CD56+ cells were more sensitive to stimulation with PMA and ionomycin, as they responded to this type of stimulation both in the young and the old (% and MFI analysis), in contrast to T cells, which responded to stimulation only in the young observed in both types of analysis. The expression of HSP70 was significantly higher in NKT-like cells than in T cells in the young and old groups (observed in % and MFI analysis) and additionally in the oldest group observed in MFI analysis, although the same tendency was also found in % analysis.

### Expression of SOD2 in nonstimulated and stimulated CD3+ and CD3 + CD56+ cells of the studied age groups

**Fig. 4:**
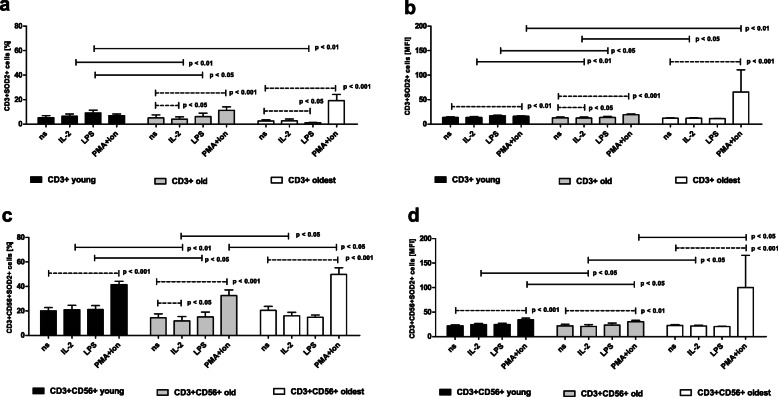
Expression of SOD2 in nonstimulated and stimulated with IL-2, LPS and PMA/ionomycin CD3+ and CD3 + CD56+ cells of the young, old and oldest age groups. Data are presented as the mean ± SEM and show the expression of SOD2 in the studied cell populations demonstrated as the percentage of cells with the expression of SOD2 protein (%) and mean fluorescence intensity (MFI). Solid horizontal lines denote statistically significant differences found between similarly treated cells of different age groups, i.e. young vs. old, young vs. oldest and old vs. oldest. Dashed horizontal lines above paired bars denote statistically significant differences between nonstimulated vs. stimulated cells within the same age group. **a.** Expression of SOD2 in CD3+ cells (%). **b.** Expression of SOD2 in CD3+ cells (MFI). **c.** Expression of SOD2 in CD3 + CD56+ cells (%). **d.** Expression of SOD2 in CD3 + CD56+ cells (MFI)

The expression of SOD2 differed enormously from the pattern presented by the other analyzed protective proteins. In nonstimulated CD3+ cells of the young age group this expression was quite low and comprised 5.32 ± 1.53% of the analyzed cells, which were insensitive to any type of stimulation (Fig. 4a). The corresponding analysis of the relative expression showed similar results, with the exception of cells stimulated with PMA/ionomycin, which showed a mild increase in SOD2 expression (Fig. 4b). The same tendency was found in CD3+ cells simulated with PMA and ionomycin in the percentage analysis; however, it was not statistically significant (Fig. 4a). The unstimulated CD3 + CD56+ cells of the young group presented significantly higher expression of this superoxide dismutase than T cells, which comprised 19.99 ± 2.66% of the analyzed cells (*p* < 0.001). These cells showed sensitivity to stimulation with PMA/ionomycin, as detected by an over twofold increase in the percentage of NKT-like cells, revealing SOD2 expression (Fig. 4c), that, similar to T cells, was also observed in the MFI analysis (Fig. 4d).

The nonstimulated CD3+ cells of the old presented lower SOD2 expression than in the young group; i.e. 5.04 ± 2.43%, however this difference was not statistically significant. T cells responded to stimulation with PMA/ionomycin presenting an over twofold increase in this protein expression; however, after stimulation with IL-2, a mild, statistically significant decrease in SOD2 expression was observed (Fig. 4a). These data were also confirmed by MFI analysis (Fig. 4b). The unstimulated CD3 + CD56+ cells of the old group showed lower SOD2 expression than those of the young group and occurred in 14.4 ± 3.23% of the analyzed cells (*p* < 0.001), the difference, however, was not statistically significant. NKT-like cells revealed a similar pattern of SOD2 expression to T cells, with a mild decrease in cells stimulated with IL-2 and an over twofold increase in this protein expression after stimulation with PMA and ionomycin (Fig. 4c). The analysis of the relative expression confirmed the increase in SOD2 expression after stimulation with PMA/ionomycin and the tendency to lower this expression after stimulation with IL-2 (Fig. 4d).

In contrast to the other analyzed cellular protective proteins in nonstimulated CD3+ cells of the oldest, SOD2 expression was low and involved 2.55 ± 0.98% of the analyzed cells. T cells of the oldest seniors stimulated with PMA and ionomycin showed a nearly 7.5-fold increase in SOD2 expression; however, after stimulation with LPS, these cells revealed an over twofold decrease in the expression of SOD2 (Fig. 4a). The increase after stimulation with PMA/ionomycin and the tendency to decrease after stimulation with LPS were also observed when the analysis of the relative expression was performed (Fig. 4b). The unstimulated CD3 + CD56+ cells of the oldest revealed significantly higher expression of SOD2 protein than CD3+ cells; i.e. 20.41 ± 3.19% of the analyzed cells (*p* < 0.001). These cells also revealed sensitivity to stimulation with PMA and ionomycin, which resulted in an over twofold increase in SOD2 expression (Fig. 4c), which was also confirmed by MFI analysis (Fig. 4d).

To summarize, the analysis of the SOD2 expression pattern showed that it differed from the other protective proteins. In contrast to SIRT1 and HSP70, all age groups were sensitive to the process of stimulation with PMA and ionomycin, including the oldest seniors. Intriguingly, IL-2 did not induce a significant increase in the expression of this protein in the analyzed groups. The expression of SOD2 was significantly higher in NKT-like cells than in T cells in all studied age groups.

### Relationships between the studied parameters of CD3+ and CD3 + CD56+ cells stimulated under the same conditions

Correlation analysis was performed for parameters studied in nonstimulated and stimulated CD3+ and CD3 + CD56+ cells cultivated in vitro for 48 h. The cells were treated with IL-2, LPS and PMA with ionomycin. The analysis revealed strong positive correlations (the respective symbols correspond to *p* values: * < 0.05, ** < 0.01, *** < 0.001) between the expression of cellular protective proteins, i.e. SIRT1 and HSP70 in both nonstimulated, cultured for 48 h control cells, i.e. T lymphocytes (Rs = 0.908*** in % analysis and Rs = 0.945*** in MFI analysis) and NKT-like cells (Rs = 0.883*** in % analysis and Rs = 0.921*** in MFI analysis) and stimulated with IL-2, LPS and PMA with ionomycin CD3+ cells (Rs range in % analysis: 0.915*** - 0.931***; Rs range in MFI analysis: 0.880***- 0.957***) and CD3 + CD56+ cells (Rs range in % analysis: 0.813***- 0.926***; Rs range in MFI analysis: 0.831***- 0.858***).

### Correlations between age and studied parameters in nonstimulated or stimulated under the same conditions CD3+ and CD3 + CD56+ cells

Some relationships between cellular protective proteins and the age of donors were found in the correlation analysis performed for T lymphocytes and NKT-like cells cultivated 48 h in vitro, which concerned both unstimulated, control cells and cells stimulated with IL-2, LPS and PMA with ionomycin. Interestingly, the expression of SIRT1 and HSP70 showed similar, rather weak and only sometimes moderate positive correlations with age in both CD3+ and CD3 + CD56+ cells that were unstimulated or stimulated with LPS or PMA with ionomycin. They were observed in the MFI analysis and confirmed in most cases by the analysis of the percentages of positive cells. These correlations were found neither for SIRT1 nor for HSP70 expression in both T and NKT-like cells stimulated with IL-2. They were not found also for HSP70 expression in NKT-like cells stimulated with PMA and ionomycin (Table 1).

**Table 1:** Correlation analysis concerning the relationships between age and the expression of cellular protective proteins

CD3+ cells							
**Compared parameters**							
**Parameter**	**Stimulation type**	**SIRT1 [%]**	**SIRT1 [MFI]**	**SOD2 [%]**	**SOD2 [MFI]**	**HSP70 [%]**	**HSP70 [MFI]**
**Age**	**none**	0.352***	0.307**	Ns	Ns	0.277*	0.287**
	**IL-2**	Ns	Ns	Ns	Ns	Ns	Ns
	**LPS**	0.433***	0.414***	−0.327**	−0.231*	0.367***	0.392***
	**PMA/ion**	Ns	0.396***	Ns	0.25*	Ns	0.242*
**CD3 + CD56+ cells**							
**Compared parameters**							
**Parameter**	**Stimulation type**	**SIRT1 [%]**	**SIRT1 [MFI]**	**SOD2 [%]**	**SOD2 [MFI]**	**HSP70 [%]**	**HSP70 [MFI]**
**Age**	**none**	0.353***	0.297**	Ns	Ns	0.269*	0.233*
	**IL-2**	Ns	Ns	Ns	Ns	Ns	Ns
	**LPS**	0.382***	0.383***	Ns	Ns	0.345**	0.39***
	**PMA/ion**	Ns	0.242*	Ns	Ns	Ns	Ns

All values are presented as statistically significant Spearman’s correlation coefficients (Rs). ‘Ns’ denotes statistically not significant, the respective symbols denote * < 0.05, ** < 0.01, *** < 0.001

SOD2 expression revealed a weak, negative correlation with age only for CD3+ cells stimulated with LPS, as confirmed in both types of analysis (MFI and %). The only weak positive correlation between SOD2 expression and age was observed in CD3+ cells treated with PMA and ionomycin in MFI analysis, which was not confirmed in the analysis of the percentages of positive cells (Table 1).

## Discussion

Adaptive homeostasis is a highly conserved process when cells activate various signaling pathways in response to detrimental internal or external factors. It usually generates changes in gene expression and stress resistance to counteract stressful conditions. Cellular protective proteins involved in the heat shock response and antioxidant protection play a crucial role in this process [5]. Ageing of the immune system is characterized both by increased inflammation and a decreased immune response mastered, especially by the innate immune system [60]. The adaptive immune system in the ageing process is characterized by a decrease in naive T cell numbers, which results from thymic involution and leads to shrinking of the TCR repertoire on one side and an increase in memory T cell numbers on the other side. Decreases in regulatory B cell numbers and immunoglobulin production are also observed. During ageing, immune cell homeostasis is disturbed; however, these changes are not always detrimental but may also be adaptive or remodelling of the immune system [4]. Thus, the presented study might provide some knowledge concerning these more adaptive aspects of immune ageing.

Data presented in the study concerned T and NKT-like cells cultured 48 h in vitro in the absence (nonstimulated, control cells) or presence of stimulating agents; i.e. IL-2, LPS and PMA with ionomycin. When the obtained results were compared to data received for T and NKT-like cells originating from whole blood samples analyzed shortly after their collection [45], it appeared that although some similarities were observed in the general expression pattern of the studied cellular protective proteins, there were also noticeable differences. SIRT1 analysis showed that in both nonstimulated T and NKT-like cells cultivated for 48 h, the in vitro expression of this protein was at least a few times higher than that in cells analyzed directly after blood sample collection [45]. These results correspond to our previous observations made for NK cells analyzed shortly after blood sample collection and after 48 h of cell culture, which revealed an increase in the level of oxidative stress in cultured cells estimated by the measurement of carbonyl groups and 8-isoprostane concentrations in NK cell extracts [46]. Cell culture conditions usually promote the development of oxidative stress, which was thoroughly discussed by Professor Barry Halliwell [61]. Simultaneously, we found in our studies that higher expression of SIRT1 protein in CD3 + CD56+ cells compared to CD3+ cells observed shortly after blood sample collection [45] was also maintained in the cell culture.

In contrast, when the expression of HSP70 and SOD2 in cultured CD3+ and CD3 + CD56+ cells shown in the present study was compared to the results obtained for whole blood samples [45], it appeared that higher expression of these protective proteins was observed in lymphocytes analyzed shortly after blood sample collection [45]. The expression of HSP70 and SOD2 in both unstimulated T and NKT-like cells cultivated in vitro in the present study was a few times lower. Similar to SIRT1 expression, in both HSP70 and SOD2 analyses, higher expression of these protective proteins in CD3 + CD56+ cells compared to CD3+ cells observed directly after blood sample collection was also maintained in cells cultivated in vitro. This higher expression observed in cells from whole blood samples may result from a specific gene expression pattern that identifies the studied protective proteins. Genes for both HSP70 and SOD2 are expressed shortly after cell activation, which was shown in resting and stimulated NK and NK-92 cells [62, 63]. This phenomenon may result from the activation of heat shock factor-1 within minutes after the appearance of the stress factor and an increase in mRNA expression within 6 h after triggering a relevant signaling pathway [64, 65]. Correspondingly, an increase in SOD2 mRNA expression was observed within 1–2 h after stimulation [63, 66]. The process of blood sample collection and subsequent procedures might disturb cellular homeostasis and increase the expression of cellular protective proteins involved, especially in the first wave of induced transcription, including HSP70 and SOD2 [62]. In cells cultivated for 48 h in vitro, cellular homeostasis might be restored under new conditions, resulting in lower expression of these cellular protective proteins.

In contrast, SIRT1 gene expression was detected within 24 h in endothelial progenitor cells [67] and 48–96 h in HepG2 cells [68] after exposure to a stress factor. Different kinetics of this expression may result from the involvement of this gene in a rather late gradual cellular response, as was proposed for some transcription patterns [69]. Moreover, the studied cellular protective proteins are involved in different signaling pathways that are under the control of various cellular factors, so regulatory mechanisms might be more complex [70, 71].

However, the present study not only provides some novel data concerning the role of the adaptive stress response in adaptation to changing environmental conditions but also shows the influence of the process of cell stimulation on the expression of cellular protective proteins involved in the maintenance of this response in T lymphocytes and NKT-like cells.

Interestingly, when cultured for 48 h, both T lymphocytes and NKT-like cells of the oldest seniors revealed increased and constant, insensitive to further stimulation expression of SIRT1 and HSP70. These data correspond to the results described by us previously for cultured NK cells and their subpopulations CD56dim and CD56bright cells [46, 47]. The findings described earlier in studies concerning human monocytes and lymphocytes [18, 72] might indicate the involvement in the process of ageing more common mechanisms of the cellular adaptive response. They were also confirmed by a strong positive correlation found between the SIRT1 and HSP70 expression levels observed for these proteins in NKT-like and T cells in the present study and in our previous study regarding the analysis performed shortly after blood sample collection [45]. Analogous relationships were observed for NK cells when these parameters were analyzed in whole blood samples shortly after sample collection [44] and cells cultured in vitro with or without stimulation [46]. Both SIRT1 and HSP70 are vitagenes crucial for the maintenance of cellular homeostasis and activation of the cellular stress response. This process is under the control of a family of heat shock transcription factors, which under normal conditions are expressed and maintained in an inactive state [71]. SIRT1 was discovered by Westerheide et al. to directly deacetylate HSF1 and maintain HSF-1 in a state competent for DNA binding to enable transcription of heat shock genes to produce molecules with antioxidant and antiapoptotic activities [71, 73]. This relationship may explain the strong positive correlation found between SIRT1 and HSP70 in our studies.

The expression pattern of SIRT1 and HSP70 in CD3+ and CD3 + CD56+ cells of the young and the elderly differed from that observed in the oldest seniors. T cells of these two age groups were more sensitive to stimulation with IL-2 regarding both SIRT1 and HSP70 expression as they responded to this stimulating factor both in the young and the old. NKT-like cells appeared to be more sensitive to stimulation with PMA and ionomycin regarding both HSP70 and SIRT1 expression. HSP70 expression was increased both in the young and in the old age group and SIRT1 expression was increased only in the young group, in contrast to T cells, which revealed increase in the young group only and revealed no increase after stimulation, respectively. Interestingly, when these results were compared to our data obtained earlier for NK cells cultured for 48 h in similar control and stimulatory conditions, NK cells were characterized by even higher sensitivity to stimulation than that observed in T or NKT-like cells and responded to stimulation by both IL-2 and PMA and ionomycin [46].

The SOD2 expression pattern after stimulation differed in the present study from the other protective proteins. In contrast to SIRT1 and HSP70, both T and NKT-like cells in all age groups were sensitive to the process of cell activation with PMA and ionomycin, including the oldest seniors. Intriguingly, IL-2 did not stimulate an increase in SOD2 expression in the studied age groups in either T or NKT-like cells. The SOD2 expression pattern of both lymphocyte populations was similar to that found by us earlier in cultured NK cells, although NK cells also responded to IL-2 stimulation to some extent [46]. However, in CD3 + CD56+ and CD3+ cells, the IL-2 signaling pathway seemed to be not involved in the stimulation of SOD2 expression. Interestingly, NKT-like cells showed significantly higher levels of SOD2 expression than T cells. Similar results were obtained for T and NKT-like cells analyzed from whole blood samples shortly after sample collection [45].

Some differences in the sensitivity to the process of stimulation observed between T and NKT-like cells may result from various expression of cell surface receptors involved in the relevant signaling pathways. There are three classes of IL-2 receptors: low affinity receptors composed of alpha chains (CD25) only (IL-2Rα receptors), intermediate affinity receptors composed of two subunits, β (CD122) and γ chains (IL-2Rβγ receptors), and high affinity receptors composed of three subunits, the α chain responsible for IL-2 binding and β and γ chains involved in signal transduction (IL-2Rαβγ receptors). Both IL-2Rβγ and IL-2Rαβγ receptors are active in IL-2 signaling [49, 74]. NKT-like and NK cells were described to differ in the number and type of IL-2 receptors on the cell surface. It was found that the IL-2Rα chain, the CD25 molecule, was expressed by 27% of CD3+ cells, only 7% of CD3 + CD56+ cells and 2% of CD3-CD56+ cells (NK cells). In contrast, the IL-2Rβ chain, a CD122 molecule, was constitutively expressed by 99% of NK cells, 74% of CD3 + CD56+ cells and only 10% of CD3+ cells. Moreover, the intensity of staining for CD122 was 10-fold higher on NK cells than on NKT-like cells [75]. Thus, the sensitivity of immune cells to IL-2 depends on the types of IL-2Rs expressed on their cell surface and the inductive versus constitutive expression of the different IL-2R chains [74].

Furthermore, some changes in IL-2R expression were also observed in the process of ageing, including a decrease in the expression of receptors for IL-2 on the cell surface of T cells [76], an increase in the expression on the surface of CD4 naive T cells [77] and no changes on the surface of NK cells [78]. These differences in IL-2R expression may explain the highest sensitivity to IL-2 stimulation observed in the young we found especially in SIRT1 and HSP70 expression in both T and NKT-like cells. The stimulatory effect of IL-2 on SIRT1 and HSP70 expression in the old age group was observed only in T cells, which might be caused by higher expression of the CD25 molecule on the surface of T cells and its involvement in IL-2 signaling [78]. The stimulatory effect of IL-2 on SIRT1 and HSP70 expression disappeared in the oldest seniors, which might result from decreasing with age expression of IL-2 receptors on the cell surface on one side [76] and development of an adaptive stress response with increased expression of cellular protective proteins both in T and NKT-like cells on the other side. However, in the correlation analysis performed between age and the relevant protective protein expression, no detectable associations were found. Furthermore, IL-2 was not found to stimulate T cells or NKT-like cells to increase the expression of SOD2. The influence of age and various expressions of receptors on the cell surface might then be counterbalanced by other regulatory mechanisms involved in JAK/STAT, RAS/MAPK and PI3K/AKT pathways mediated by IL-2 signaling [79, 80].

The ageing process is also associated with a decreasing function of TLRs resulting from their impaired downstream signalling [81, 82]. LPS treatment and activation of the TLR4 signaling pathway were found to elicit SIRT1 [83], HSP70 [84] and SOD2 [85] expression. However, in our studies, stimulation with LPS did not cause significant changes in the expression levels of the analyzed protective proteins. The changes were observed only in T cells of the oldest seniors and concerned a significant, over twofold decrease in SOD2 expression representing alterations that might characterize the process of ageing. In NKT-like cells of the oldest, a similar tendency was observed; however, it was not statistically significant.

PMA used for nonspecific stimulation of lymphocytes is an analog of diacylglycerol, a key mediator of numerous signaling pathways. Ionomycin stimulates calcium release from the endoplasmic reticulum, activates calcium sensitive enzymes and acts synergistically with PMA [86]. The signaling machinery involves signaling pathways upstream of the transcription factors NFAT, AP-1 and NF-κB. TCR activation triggers signaling to phospholipase C, which cleaves phosphoinositol phosphate to generate two crucial signaling molecules, i.e. inositol phosphate involved in initiation of the NFAT signaling pathway and diacylglycerol associated with activation of the protein kinase C (PKC)–dependent induction of IκB kinase (IKK) and the Ras/Raf/MAPK signaling pathways, leading to activation of both NF-κB and AP-1 transcription factors [87].

PMA then stimulates the activity of distinct transcription factors involved in various signaling pathways. A positive feedback loop between SIRT1 expression and NF-κB has been found [88]. NF-κB and AP-1 were also reported to be transcription factors that play important roles in the regulation of both constitutive and inductive expression levels of superoxide dismutases, including SOD2 [89]. Thus, stimulation of both T and NKT-like cells with PMA and ionomycin in our studies resulted in a strong response observed, especially for SOD2 expression, in all age groups. When HSP70 and SIRT1 expression was analyzed, NKT-like cells appeared to be more sensitive to stimulation with these factors than T cells. Interestingly, CD3+ and CD3 + CD56+ cells of the oldest seniors developed an adaptive stress response in regard to SIRT1 and HSP70 but not SOD2 expression. These observations indicate the involvement of different signaling pathways in the activation of various cellular protective proteins by immune modulators [70, 71].

The analysis of correlations between age and the expression of cellular protective proteins revealed that SIRT1 and HSP70 correlated positively with age in most analyzed conditions of stimulation in CD3+ cells and CD3 + CD56+ cells, although in NKT-like cells, they were slightly lower. A remarkable age-related increase in SIRT1 protein expression was also observed earlier by Kilic and coworkers in human serum samples [29]. Njemini et al. found a general age-dependent rise in the basal level of HSP70 and other types of HSP proteins (HSP32, HSP90) in monocytes and lymphocytes [17]. These relationships may result indirectly from a strong association between SIRT1 and HSP70, which was also observed in our studies and corresponded to experiments performed by Westerheide et al. [73]. This connection characterizes the adaptive stress response that develops during the process of ageing [90].

Although ageing is usually associated with an increase in oxidative stress and stimulation of SOD2 antioxidant defence [91], we found a negative correlation between age and manganese superoxide dismutase expression in CD3+ cells treated with LPS, similar to the results of our previous studies on NK cells and their subpopulations cultivated in vitro for 48 h [46, 47]. We found that the lowest concentration of oxidative stress markers, i.e. carbonyl groups and 8-isoprostanes was observed in NK cells of the oldest seniors. Intriguingly, the highest concentrations of these oxidative stress markers were detected in the young, although all the results were still within a normal range. This phenomenon may explain the negative correlation between SOD2 and age in our studies. Interestingly, it corresponded to an even higher negative correlation between age and carbonyl group and 8-isoprostane concentrations found in both nonstimulated and stimulated NK cells [46]. This uncommon decrease in oxidative stress levels observed in the oldest seniors may result from mitohormesis, a phenomenon described by Ristow and Zarse. They noted that ROS production in the mitochondria may cause the development of a mitochondrial adaptive response to promote stress resistance and long-term reduction of oxidative stress [92].

In contrast, a positive correlation observed between SOD2 expression in CD3+ cells and age in samples stimulated with PMA and ionomycin may result from a strong and increasing with age cell activation process with these stimulating factors we found in the current study.

Interestingly, in both NKT-like and T cells analyzed directly after blood sample collection, the observed relationships were higher compared to cells cultured in vitro and presented in the current study, which might be caused by differences in the expression levels of protective proteins found in the cultured cells and cells analyzed shortly after collection [45]. Similar to NKT-like and T cells, associations between age and the expression of corresponding protective proteins were found in NK cells, and they were also higher in cells from whole blood samples than in the cell culture [44, 46]. The observed phenomena indicated the involvement of similar adaptive mechanisms in the spectrum of T-cells, NK cells and NKT-like cells.

## Conclusions

The oldest seniors developed an adaptive stress response in both T and NKT-like cells regarding the expression of SIRT1 and HSP70, which was increased and insensitive to further stimulation, in contrast to SOD2, that showed a more inducible pattern of expression. NKT-like cells revealed higher than T cells expression levels of cellular protective proteins in both stimulated and control, nonstimulated cells and showed a distinct sensitivity to stimulation compared to T cells, dependent on the type of a stimulatory factor. T lymphocytes revealed higher sensitivity to IL-2 regarding SIRT1 and HSP70 expression, and NKT-like cells were more sensitive to stimulation with PMA and ionomycin concerning the expression of these proteins, indicating cell type-specific differences in the relevant signaling pathways.

## Abbreviations

ADL: Activities of daily living
AMPK: 5’AMP-activated protein kinase
AP-1: Activator protein 1
CRP: C reactive protein
eNOS: Endothelial nitric oxide synthase
FOXO: Forkhead transcription factor class O
FSC: Forward scatter
HSF: Heat shock factor
HSP: Heat shock protein
IL-2: Interleukin 2
IL-2R: Interleukin-2 receptor
IP3: Inositol triphosphate
LPS: Lipopolysaccharide
MFI: Mean fluorescence intensity
MMSE: Mini Mental State Examination
NAD: Nicotinamide adenine dinucleotide
NFAT: Nuclear factor of activated T-cells
NF-κB: Nuclear factor kappa-light-chain-enhancer of activated B cells
NKR: NK cell receptor
NSAID: Nonsteroid anti-inflammatory drug
PBMCs: Peripheral blood mononuclear cells
PGC: Peroxisome proliferator - activated receptor gamma coactivator
PKC: Protein kinase C
PMA: Phorbol myristate acetate
PPAR-γ: Peroxisome proliferator-activated receptor gamma
ROS: Reactive oxygen species
Rs: Spearman’s rank correlation coefficient
SIRT: sirtuin
SOD: Superoxide dismutase
SSC: Side scatter
TCR: T cell receptor
TLR: Toll-like receptor
